# *Mycobacterium avium* in Community and Household Water, Suburban Philadelphia, Pennsylvania, USA, 2010*–*2012

**DOI:** 10.3201/eid2503.180336

**Published:** 2019-03

**Authors:** Leah Lande, David C. Alexander, Richard J. Wallace, Rebecca Kwait, Elena Iakhiaeva, Myra Williams, Andrew D.S. Cameron, Stephen Olshefsky, Ronit Devon, Ravikiran Vasireddy, Donald D. Peterson, Joseph O. Falkinham

**Affiliations:** Lankenau Medical Center and Lankenau Institute for Medical Research, Wynnewood, Pennsylvania, USA (L. Lande, R. Kwait, R. Devon, D.D. Peterson);; University of Manitoba, Winnipeg, Manitoba, Canada (D.C. Alexander);; The University of Texas Health Science Center, Tyler, Texas, USA (R.J. Wallace, Jr., E. Iakhiaeva, R. Vasireddy);; Virginia Polytechnic Institute and State University, Blacksburg, Virginia, USA (M. Williams, J.O. Falkinham, III);; University of Regina, Regina, Saskatchewan, Canada (A.D.S. Cameron, S. Olshefsky)

**Keywords:** Nontuberculous mycobacteria, *Mycobacterium avium*, household biofilms, VNTR, *Mycobacterium avium* water, genome sequencing, Philadelphia, Pennsylvania, United States, tuberculosis and other mycobacteria, bacteria

## Abstract

Attention to environmental sources of *Mycobacterium avium* complex (MAC) infection is a vital component of disease prevention and control. We investigated MAC colonization of household plumbing in suburban Philadelphia, Pennsylvania, USA. We used variable-number tandem-repeat genotyping and whole-genome sequencing with core genome single-nucleotide variant analysis to compare *M. avium* from household plumbing biofilms with *M. avium* isolates from patient respiratory specimens. *M. avium* was recovered from 30 (81.1%) of 37 households, including 19 (90.5%) of 21 *M. avium* patient households. For 11 (52.4%) of 21 patients with *M. avium* disease, isolates recovered from their respiratory and household samples were of the same genotype. Within the same community, 18 (85.7%) of 21 *M. avium* respiratory isolates genotypically matched household plumbing isolates. Six predominant genotypes were recovered across multiple households and respiratory specimens. *M. avium* colonizing municipal water and household plumbing may be a substantial source of MAC pulmonary infection.

Nontuberculous mycobacteria (NTM) are opportunistic human pathogens. Several species of NTM, including members of the *Mycobacterium avium* complex (MAC), can cause potentially life*-*threatening pulmonary infections that are difficult to treat (*1**,**2*). In 1989, Prince et al. described MAC pulmonary disease in persons without predisposing conditions (*3*). That study consisted of 21 patients from 2 hospitals in greater Philadelphia, Pennsylvania, USA, 1 of which was Lankenau Medical Center, located in Montgomery County. In 2012, Adjemian et al. identified Montgomery County as 1 of 7 US counties associated with a high risk for MAC lung disease (*4*). The reason for this risk was not apparent.

A recognized source of NTM infections is the environment (5*–*7). Many species of NTM are found in drinking water distribution systems (*8*), buildings (*9*), and household plumbing (*7**,**10*). Pulmonary NTM disease often recurs, even after completion of prolonged courses of therapy and periods of NTM*-*free sputum cultures (*11*). In a molecular epidemiologic study that confirmed transmission of *M. avium* from potable water to patients, the same strain of *M. avium* was found in 2 groups of patients with AIDS and in the recirculating hot water systems of the 2 hospitals at which they had been treated (*12*). To prevent NTM infection and reinfection of vulnerable populations, identification and elimination of environmental reservoirs is crucial. Remarkably, however, establishing epidemiologic links between clinical NTM isolates and specific environmental reservoirs has been difficult, partly because of the absence of robust tools for comparison of NTM isolates.

Newer molecular techniques, including variable-number tandem-repeat (VNTR) genotyping and whole-genome sequencing (WGS), provide greater discrimination of MAC isolates than previous methods (*13**–**15*). In this study, we used VNTR and WGS to test the hypothesis that household plumbing is a reservoir for NTM and is responsible for some cases of pulmonary MAC infection.

## Materials and Methods

### Study Setting and Population

 Lankenau Medical Center is a community-based, academic medical center in Montgomery County, Pennsylvania, adjacent to Delaware and Philadelphia Counties. We prospectively identified and randomly chose study participants from patients at Lankenau Medical Center for whom MAC pulmonary infection was newly diagnosed during 2010*–*2012. Because the study was designed to investigate MAC pulmonary disease among women, we excluded male patients from the study. Chest computed tomography images were reviewed by the principal investigator (L.L.) and by a chest radiologist experienced in bronchiectasis (*16*). All patients had evidence of nodular disease and bronchiectasis. Potential participants for whom >2 sputum cultures or 1 bronchoscopic culture were positive for MAC were investigated further. Patients who met the microbiological, radiographic, and clinical criteria for MAC as outlined by the 2007 American Thoracic Society/Infectious Disease Society of America (ATS/IDSA) Statement on Nontuberculous Lung Disease (*2*) were offered study enrollment. Clinical and demographic information was obtained through chart review and from questionnaires that were filled out by patients with the aid of study personnel (Table 1).

**Table 1:** Characteristics of patients with *Mycobacterium avium* complex infection and study controls, Philadelphia, Pennsylvania, USA, 2010*–*2012*

Characteristic	Patients, n = 26	Controls, n = 11
Age, y		
40–49	1	4
50–59	2	0
60–65	4	3
66–69	3	1
70–75	3	2
76–79	2	1
>80	11	0
Median (range)	77 (44–90)	64 (40–77)
Race		
White	25 (97)	11 (100)
Black	1 (3)	0
Tobacco use		
Lifetime nonsmoker	15 (58)	8 (73)
Current smoker	0	0
Former smoker	11 (42)	3 (27)
Pack-years, no.		
5–10	4	0
11–20	4	2
21–30	1	1
31–40	0	0
>40	2	0
Preexisting lung disease†		
Present	3 (12)	3 (27)
Absent	23 (88)	8 (73)

*Values are no. (%) except as indicated. Only female patients were included in the study. †Lung disease recognized before diagnosis of nontuberculous mycobacteria infection.

The study protocol was approved by the Main Line Health Institutional Review Board, and informed consent was obtained from participants before study inclusion. Microbiology components of the study performed at partner institutions were deemed exempt from institutional review board review, and patient identifiers were removed from cultures or DNA samples before evaluation. 

### Control Population

For controls, we included 11 geographically matched persons from households serviced by the same municipal water system as the patients. Control participants were healthy neighbors of MAC patients and persons with bronchiectasis who had negative MAC culture results. All controls volunteered to have samples from their homes cultured (Figure).

**Figure:**
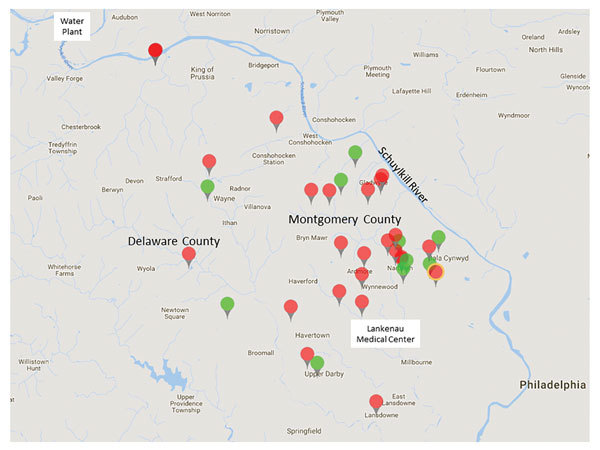
Area of study of *Mycobacterium avium* in community and household water, Philadelphia, Pennsylvania, USA, 2010*–*2012. The 26 patients (black tags) and 11 controls (gray tags) lived in suburban Philadelphia (Montgomery and Delaware Counties). White dot symbol in upper left indicates 3 patients who lived in the same apartment building. Star indicates patient and control households in very close proximity. All households were supplied by water that came from the Schuylkill River and was processed by the same water treatment plant.

### Isolation and Identification of MAC

Isolation and identification of MAC from household biofilms were performed as described by Falkinham et al (*6**,**7*). We chose biofilm samples because the numbers of NTM are higher in biofilms than in water (*7*). Swab samples were vortexed in a tube for 60 s, then 0.1 mL of the suspended cells was spread in triplicate on Middlebrook 7H10 agar (Becton Dickinson and Company, https://www.bd.com) containing 0.5% glycerol and 10% oleic acid–albumin, sealed with Parafilm (Bemis NA, http://www.bemis.com), and incubated at 37°C. At weekly intervals, the plates were examined for evidence of mycobacterial colonies. Putative mycobacterial colonies were picked and streaked on M7H10 agar, and identical colonies on the original isolation M7H10 agar plates were counted to calculate CFUs per square centimeter of surface. After growth, colonies were acid-fast stained, DNA was isolated, and colonies were identified by PCR amplification and restriction digestion fragment pattern analysis of the *hsp-65* gene. MAC were identified to species level by partial 16S rRNA gene sequencing, as previously described (*13**,**17*). *M. avium* isolates were subjected to PCR for IS*901*, and isolates negative for IS*901* were classified as *M. avium* subsp. *hominissuis* (*18**,**19*).

### Respiratory Samples

Respiratory samples were processed at the Lankenau Medical Center Microbiology Laboratory by use of standard methods, including a commercial hybridization assay (AccuProbe; Hologic, Inc., http://www.hologic.ca). For each patient for whom respiratory culture was positive, 1 MAC isolate was subcultured and sent for further analysis to the University of Texas Health Science Center (Tyler, TX, USA) and Virginia Polytechnic Institute and State University (Blacksburg, VA, USA) (Table 2). Species identification was performed by the same methods used for biofilm isolates.

**Table 2:** Description of MAC isolates from respiratory and household samples, Philadelphia, Pennsylvania, USA, 2010*–*2012*

Patient	Case no.	Respiratory sample MAC species (VNTR type)	Household (biofilm) sample			VNTR match between patient's respiratory and own household samples	VNTR match between patient's respiratory and community household samples
			No. sites sampled	No. (%) sites positive for *M. avium*	VNTR type(s)		
1	P1	*M. chimaera*	13	6 (46.1)	22 and 37a[B9]	NA	NA
2	P2	*M. avium* (15)	11	1 (9.1)	47	No	No
3	P4	*M. avium* (14a)	8	1 (12.5)	14a	Yes	Yes
4	P5	*M. avium *(7)	15	0	NA	NA	No
5	P6	*M. avium* (36)	6	4 (66.7)	14a	No	Yes
6	P7	*M. avium* (37a[B1])	13	2 (15.3)	14a	No	Yes
7	P8	*M. avium* (14a)	8	4 (50.0)	14a	Yes	Yes
8	P9	*M. avium* (36)	11	2 (18.1)	14a	No	Yes
9	P10	*M. avium* (22)	13	8 (61.5)	14a, 22, and 37a[B1]	Yes	Yes
10	P11	*M. avium *(36)	10	8 (80.0)	14a, 31a, 36, and 37a[B2]	Yes	Yes
11	P12	*M. intracellulare*			NA	NA	NA
12	P13	*M. avium *(14a)	7	2 (28.5)	14a and 37a[B1]	Yes	Yes
13	P14	*M. avium *(14a)	10	3 (30.0)	14a	Yes	Yes
14	P18	*M. avium *(37)	11	4 (36.3)	14a, 31a, 36 and 37	Yes	Yes
15	P19	*M. avium *(14a)	6	1 (16.7)	31a	No	Yes
16	P20	*M. intracellulare*	6	3 (50.0)	37	NA	NA
17	P21	*M. avium *(14a)	8	1 (12.5)	14a	Yes	Yes
18	P22	*M. intracellulare*	10	3 (30.0)	38a	NA	NA
19	P23	*M. avium* (14a)	5	5 (100)	31a and 36	No	Yes
20	P24	*M. avium* (14a)	8	2 (25.0)	14a	Yes	Yes
21	P27	*M. avium* (37)	13	3 (23.0)	36	No	Yes
22	P28	*M. avium *(37a[B2])	6	2 (33.3)	30 and 36	No	Yes
23	P30	*M. avium *(55)	14	0	NA	No	No
24	P32	*M. chimaera*	15	0	NA	NA	NA
25	P33	*M. avium* (14a)	4	1 (25.0)	14a	Yes	Yes
26	P34	*M. avium *(14a)	19	5 (26.3)	14a	Yes	Yes
Control							
27	P3	No MAC	9	0	NA	NA	NA
28	P17	No MAC	5	4 (80.0)	14a and 36	NA	NA
29	P26	NA	14	4 (28.5)	36	NA	NA
30	P29	No MAC	10	3 (30.0)	31a and 36	NA	NA
31	P31	NA	3	0	NA	NA	NA
32	P35	NA	4	1 (25.0)	19	NA	NA
33	P36	NA	11	5 (45.4)	31a and 36	NA	NA
34	P37	NA	6	3 (50.0)	36	NA	NA
35	P38	NA	9	1 (11.1)	30	NA	NA
36	P40	NA	9	3 (33.3)	14a and 37a[B1]	NA	NA
37	P41	NA	4	0	NA	NA	NA
Total			334	95 (28.4)			

*ID, identification; MAC, *Mycobacterium avium* complex; NA, not applicable; VNTR, variable-number tandem-repeat.

### Household Samples

Samples for mycobacterial culture were collected from all patient and control households (Table 2). Study personnel used sterile swabs to sample surfaces in contact with water, including kitchen plumbing (sink faucets, refrigerator ice and water dispensers), bathroom plumbing (shower pipes, showerheads, tub and sink faucets, toilet water tanks), and household humidifiers attached to central heating units. After sampling, each swab was placed in 3 mL of water from the source being tested, sealed in a sterile conical tube, and sent to the Falkinham Lab at Virginia Polytechnic Institute and State University. When cultures from a sample included colonies of diverse morphology, those colonies were counted, and a representative colony of each morphotype suspected of being an NTM was selected for analysis.

### Genotyping of *M. avium* Isolates

VNTR genotyping of *M. avium* isolates was performed by using 6 previously characterized *M. avium* tandem-repeat sequences (MATR1, MATR2, MATR3, MATR7, MATR13, MATR14) (*20*) plus mycobacterial interspersed repetitive unit locus 3 (*21*). Internal transcribed spacer (ITS) sequencing (*17**,**22*) was also performed for all *M. avium* isolates. Results were compared with an in-house database that included genotyping results for 416 *M. avium* subsp. *hominissuis* isolates from 121 patients and 80 biofilm samples (*18*). Genotypes were assigned as previously published for *M. avium* subsp. *hominissuis* (*13**,**18**,**23*). New numbers were assigned to VNTR/ITS combinations not previously encountered (R.J. Wallace, Jr., unpub. data). VNTR 37a strains were further subtyped by using 3′ *hsp65* sequencing (*24*) (Appendix Table 1).

### Household Water Sources

 We collected addresses for all participants and recorded them on a regional map (Figure). All patient and control households were located in southern Montgomery County or adjacent regions of Delaware County, Pennsylvania. According to the local water utility company, these areas of Montgomery and Delaware Counties are serviced by 1 water treatment plant, which processes surface water from the Schuylkill River and its tributaries (http://www.montcopa.org/DocumentCenter/View/4342). The age of the water pipes leading to the households ranged from 65 to 115 years. Most (65%) homes were >50 years old.

### WGS

To assess genomic diversity within the most common VNTR types and the genetic similarity of respiratory and plumbing isolates from individual households, we performed WGS. We selected 40 *M. avium* isolates, representing genotypes 14a, 36, 22, and 37a (subtypes B1 and B2) (Appendix Table 2). Data associated with this study have been registered in the National Center for Biotechnology Information database as BioProject ID PRJNA339271 (https://www.ncbi.nlm.nih.gov/bioproject/339271). *M. avium* was isolated from the respiratory tract of 17 participants, from the respiratory tract and >1 household plumbing source for 12 of these 17, and from household plumbing only of the other 4 participants. A final participant was represented by 2 subcultures of a single respiratory isolate. These 2 biological replicates were processed separately and included to assess genomic variation that might be introduced during strain manipulation (e.g., subculture, DNA extraction, library preparation, and sequencing). Methods for WGS and bioinformatic analyses have been described (*25*). In brief, we generated paired-end libraries with the NEBNext Ultra DNA library prep kit and NEBNext Multiplex Oligos for Illumina (New England BioLabs, https://www.neb.ca). WGS was performed on an Illumina MiSeq platform by using the MiSeq reagent version 3 kit (600 cycle) according to the manufacturer’s guidelines (Illumina, Inc., https://www.illumina.com). High-quality core single-nucleotide polymorphism comparison was performed by using SNPhyl version 1.0.1 (*26*). Isolates from the same cluster were considered epidemiologically related if they differed by <15 single-nucleotide variants (SNVs) (*27*).

## Results

### Patient Characteristics

 All 26 patients were female, had nodular bronchiectasis confirmed by computed tomography, and had MAC lung disease as defined by the ATS/IDSA criteria (*2*). At enrollment, patient median age was 77 years (range 44*–*90 years), 97% (25/26) were white, 42% (11/26) were former smokers, and 88% (23/26) had no previous or co-occurring lung disease other than bronchiectasis (Table 1). All patients lived within 20 miles of each other (Figure): 20 in Montgomery County and 6 in Delaware County.

### Control Characteristics

All 11 control participants were female; median age was 64 years (range 40*–*77 years). Eight lacked any history or signs of pulmonary NTM disease (i.e., no cough, dyspnea, fatigue, weight loss, or recurrent respiratory infections). Three participants had bronchiectasis and >2 sputum samples that did not grow NTM on culture. Controls lived within 20 miles of each other and the patients: 7 in Montgomery County and 4 in Delaware County (Figure).

### Respiratory Samples

MAC respiratory isolates were recovered from 26 patients during the study period. The positive cultures were obtained from expectorated sputum and bronchoscopic sampling. We identified 3 species of MAC: *M. avium* subsp. *hominissuis* (21/26, 80.8%), *M. intracellulare* (3/26, 11.5%), and *M. chimaera* (2/26, 7.7%) (Table 2).

### Household Samples

We collected 334 environmental biofilm samples, including 250 samples from 26 MAC patient households (range 4–19 samples from 3–13 sites in each household) and 84 samples from the 11 control households (range 3–14 samples from 3–11 sites in each household). A total of 95 *M. avium* isolates were recovered; 30/37 (81.1%) households were positive for *M. avium*, including 22/26 MAC patient households, 19/21 *M. avium* patient households, and 8/11 control households (Table 2).

*M. avium* was recovered from the kitchen sink faucet in 21/37 (56.8%) households and from 20/37 (54.1%) of the primary bathroom sites sampled, including 15/36 (41.7%) bathroom sink faucets, 13/35 (37.1%) showerheads, and 11/29 (37.9%) shower pipes (Appendix Table 3). Among households with humidifiers connected to the central heating units (with household plumbing as their water source), 7/17 (41.1%) were positive for *M. avium.* Among other sample sites, 2/14 (14.2%) refrigerator ice/water dispensers were positive, and 0 sampled toilet tanks were positive*.*

**Table 3:** Whole-genome sequencing of selected respiratory and household *Mycobacterium avium* isolates with matched genotypes by VNTR, Philadelphia, Pennsylvania, USA, 2010*–*2012*

Case no.	Source	Genotype	SNV distance between respiratory and household isolates
P4	Kitchen sink	14a - I	51
P8	Shower pipe	14a - I	8
P10	Shower pipe	22	4
P13	Kitchen sink	14a - I	37
P14	Shower pipe	14a - I	8
P24	Refrigerator tap	14a - II	12
P24	Central humidifier	14a - II	14
P33	Kitchen sink	14a - I	24
P34	Bathroom sink	14a - I	8
P34	Kitchen sink	14a - I	11
P34	Shower pipe	14a - I	16


*SNV, single-nucleotide variant; VNTR, variable-number tandem-repeat.

### *M. avium* Genotypes

For 11/21 (52.4%) patients with *M. avium* disease, the genotype isolated from their respiratory sample was identical to the genotype recovered from their household plumbing sample (Table 2). For 18/21 (85.7%) *M. avium* patients, the respiratory isolate was the same genotype as that of plumbing biofilm isolates from >1 households within the same community. For 7/21 (33.3%) *M. avium* patients, the respiratory isolate genotype did not match that of a plumbing isolate from their own household but did match that of plumbing isolates from neighboring households. Overall, the 21 respiratory and 95 household isolates of *M. avium* encompassed 15 genotypes (Appendix Table 1). Six genotypes were broadly distributed across multiple households and respiratory specimens, and 2 genotypes were isolated from multiple household sources but not from respiratory specimens. In 11 households, the *M. avium* populations were heterogeneous, and 2–4 genotypes were recovered.

### Whole-Genome Sequences and SNV*–*Based Phylogeny

SNVPhyl analysis of *M. avium* samples identified 26,871 variable sites across the core set of 4.3 million nt positions per isolate (Appendix Table 4). WGS was performed on 8 pairs of VNTR**-**matched respiratory and household isolates. For all 8 paired samples, the distance between respiratory and household isolates of *M. avium* was 4–51 SNVs; 5 pairs were separated by <15 SNVs (Table 3). The respiratory isolate from patient 13 differed from an isolate from her own kitchen sink by 37 SNVs and by <15 SNVs from plumbing biofilm isolates from households of patient 14 (9 SNVs) and patient 4 (11 SNVs). These patients did not know one another and had not visited each other’s homes.

**Table 4:** *Mycobacterium avium* culture results for patients living in different apartments within the same apartment building, Philadelphia, Pennsylvania, USA, 2010*–*2012*

Sampling site	Culture results (VNTR type)		
Patient 9	Patient 10	Patient 11
Respiratory samples	*M. avium* (36)	*M. avium* (22)	*M. avium* (36)
Household samples			
Kitchen			
Sink faucet	*M. avium* (14a)	*M. avium* (14a)	*M. avium* (37a[B2])
Primary bathroom			
Sink faucet	No NTM	*M. chimaera*	*M. avium* (14a)
Showerhead	*M. avium* (14a)	*M. avium* (22) and *M. chimaera*	*M. avium* (37a[B2])
Shower pipe	No NTM	*M. avium* (37a[B1])	*M. avium* (37a[B2])
In-line shower filter	Not determined	Not determined	*M. avium* (31a)
Secondary bathroom			
Sink faucet	Not determined	*M. avium* (22)	*M. avium* (36)
Showerhead	Not determined	*M. avium* (37a[B1])	Not determined
Shower pipe	Not determined	*M. avium* (22)	Not determined
Humidification system			
Water inflow	No NTM	*M. avium* (22)	*M. avium* (14a)
Water in system	No NTM	*M. avium* (22)	*M. avium* (14a)
Water drainage	No NTM	Not determined	No NTM

*NTM, nontuberculous mycobacteria; VNTR, variable-number tandem-repeat.

*M. avium* patients 9, 10, and 11 resided in different apartments in the same high-rise building (Table 4). Across the 3 apartments, 32 sites were sampled and 17 *M. avium* cultures, representing 6 different genotypes, were recovered. The respiratory isolate from patient 9 was type 36, which was not found in her apartment but was the same as the respiratory isolate from patient 11 and was cultured from the apartment of patient 11. The respiratory isolate from patient 10 was type 22, which was the most common genotype recovered from her apartment (5/8 sites) and matched (i.e., 4 SNVs different) an isolate from her shower. Comparison of 5 type 14a strains isolated from sites across all 3 apartments revealed only minimal differences (25–63 SNVs). Detailed results of the WGS and SNVPhyl analyses are provided in the Appendix.

## Discussion

Determining the environmental source of infection for persons with pulmonary MAC disease has proven to be remarkably difficult. We used VNTR typing, targeted gene sequencing, and core-genome SNV analysis to compare *M. avium* isolates recovered from household plumbing biofilms with respiratory isolates from patients with MAC disease. We chose biofilm sampling of household plumbing, as opposed to direct water sampling, to enhance isolation rates of mycobacteria, which concentrate in biofilm. All households were serviced by the same water filtration plant and shared a common water source. MAC was widespread; 95 *M. avium* isolates were recovered from 22/26 (84.6%) MAC patient households (including 19/21 *M. avium* patient households) and 8/11 (72.7%) control households.

Respiratory and household isolates for 11/21 (52.4%) patients with *M. avium* nodular bronchiectatic pulmonary disease were genotypically matched. For 8 patients, the matched isolates were of the same genotype (14a). VNTR type 14a isolates were recovered from 10 (38.5%) respiratory and 16 (43.2%) household samples, and type 36 isolates were recovered from 4 (15.4%) respiratory and 10 (27%) household samples. 

A 2016 report by Iakhaeva et al. (*18*) characterized 416 *M. avium* isolates from 120 patients and 80 environmental biofilms by using the methods that we used in this study and identified 49 VNTR types/subtypes. Since then, ≈100 additional isolates have undergone VNTR typing. Despite their predominance in our study, genotypes 14a and 36 were not identified in any previous studies (*18*; R.J. Wallace Jr., unpub. data). Our previous screening of additional patients and households in nearby areas that share the same primary water source (the Schuylkill River) but different water treatment plants found VNTR types 14a and 36 in those samples as well (R.J. Wallace Jr., unpub. data.) 

VNTR genotyping is reproducible, portable, and discriminatory to the extent that isolates with different VNTR patterns are considered unrelated. However, as illustrated by the 37 and 37a subtypes found in this study, pseudoclustering can occur, such that isolates with identical VNTR patterns can have different ITS and *hsp65* sequences (*18*). Previous work with *M. tuberculosis* also demonstrates that a VNTR match is not always proof of strain identity or an epidemiologic link (*28**,**29*). To better assess the relationship between VNTR-matched isolates, we turned to core-genome SNV analysis, which included ≈4.3 million nt positions; for phylogenetic comparisons, we used 26,871 variable sites. For the most common genotype identified in this study, type 14a, strains were split into 3 distinct subclusters (Appendix Figure). Isolates from the same subcluster were similar, differing by <100 of 26,871 SNVs, even though the sequenced strains were collected over 2 years and were recovered from diverse clinical and environmental sources. This widespread distribution and persistence is consistent with extensive colonization of the local water system with >1 *M. avium* strains endemic to Montgomery and Delaware Counties. For VNTR-matched respiratory and household isolates, the maximum distance was <60 SNVs. In 5 households, <15 SNVs separated the patient respiratory isolate from a plumbing biofilm isolate, suggesting an epidemiologic association between *M. avium* infection and household water. An additional patient respiratory isolate was <15 SNVs distant from biofilm strains recovered from neighboring households. This finding suggests that MAC exposure may occur outside the patient’s home but is more likely to represent incomplete recovery of respiratory strains, household strains, or both. Consistent with incomplete recovery, respiratory isolates from 7/21 (33.3%) *M. avium* patients had genotypes that were not present in their own households but did match isolates from neighboring households. Overall, 18/21 (85.7%) *M. avium* respiratory isolates matched >1 household biofilm isolates in the same community.

Our study had several limitations. The study population was relatively small and included only women from a specific region of Pennsylvania. To assess the universality of our findings, a larger, geographically diverse cohort is required. Because of the labor-intensive nature of the study, sampling was not comprehensive for all patients or households, and it is probable that because of undersampling some patient-household matches were missed. Longitudinal studies show that over time, patients with nodular bronchiectasis usually become infected with multiple genotypes of MAC (*30*), but we evaluated only 1 respiratory sample per patient.

The WGS component was conceived as a proof-of-principle experiment and limited to 40 isolates. The results of reference-based WGS comparisons are influenced by the analysis pipeline and choice of reference genome. Stringent pipeline filters intended to eliminate mapping errors can also suppress identification of true variants (*31*). Similarly, if a divergent genome is used as a reference, only a small subset of sequencing reads will be mapped and epidemiologically relevant SNVs may be missed (*32*). However, in our study, the core genome comprised >88% of the reference genome (≈4.3 million nt) despite the inclusion of genotypically diverse strains and the conservative filters used by the SNVPhyl pipeline.

Because an epidemiologic threshold for *M. avium* has not been definitively established, it remains unclear what SNV difference constitutes a definite genetic match. In a genomic analysis of *M. abscessus*, genetic relatedness was considered probable for isolates differing by <20 SNVs and possible for isolates differing by 20–38 SNVs (*27*). A cutoff of <12–25 nt for epidemiologically related isolates has been used in other studies of mycobacteria (*33**,**34*), but determining the most suitable threshold for *M. avium* will require additional isolates and analyses. In our WGS analysis of 8 pairs of VNTR**-**matched respiratory and household isolates, 5 pairs were separated by <15 SNVs, suggesting unequivocal genetic relatedness. In the other 3 pairs, distances were 24, 37, and 51 SNVs, which may also represent genetic relatedness but cannot be definitively established. In addition, the isolate from patient 13 was <15 SNVs distant from isolates from plumbing biofilm in the households of patients 14 (9 SNVs) and 4 (11 SNVs). These patients lived in separate towns that were 3–4 miles apart and serviced by the same water company, suggesting clonal infection from a common municipal water source. With MAC disease, there is often a delay between acquisition of infection and diagnosis, during which time mutations will accumulate in the environmental reservoir and the clinical isolates. For most cases in our study, infection probably preceded sample collection by several years. The estimated mutation rate for *M. avium* ssp. *paratuberculosis* is >0.5 substitutions/genome/year (*35*), but rates may be higher for *M. avium* subsp. *hominissuis* and have shorter doubling times. Rates may also be influenced by environmental and host factors. We recently sequenced serial isolates of *M. intracellulare* from patients receiving antimicrobial drug therapy and observed mutation rates that were 25-fold higher than for *M. avium* ssp. *paratuberculosis* (0.6–1 substitutions/mo) (*25*).

Our study identified household plumbing biofilms as a reservoir for *M. avium* and suggests that local rates of MAC lung disease may be influenced by mycobacterial colonization of municipal water. We cannot rule out other reservoirs, such as soil and dust, but thus far, recovery of *M. avium* from those sites and subsequent matches to patient isolates have been minimal (*36*). Moreover, because of innate resistance to chlorine and other disinfectants typically used in water treatment (*37*), *M. avium* has a survival advantage over most waterborne pathogens. In any geographic region, the prevalence of a particular *Mycobacterium* species probably affects disease rates. A recent study in Hawaii found that *M. chimaera* was the most common NTM recovered from household plumbing and the most common NTM recovered from patients in Hawaii (*10*). The value of understanding environmental sources of MAC infection have been highlighted by recent experiences with *M. chimaera* contamination of heater–cooler units (*38*).

Proof of an environmental source of *M. avium* has broad implications regarding prevention of recurrent infection in existing patients as well as prevention of new disease in susceptible persons. Additional studies are needed to establish effective methods for eliminating environmental reservoirs of *M. avium* and other problematic NTM. Tackling this challenging problem will require engagement of water utility workers, plumbers, environmental scientists, and engineers.

Appendix Whole-genome sequencing and single-nucleotide variant–based phylogenetic analysis of *Mycobacterium avium* from community and household water, suburban Philadelphia, Pennsylvania, USA, 2010*–*2012.
