# Supplementary material for: Mycobacterium avium in Community and Household Water, Suburban Philadelphia, Pennsylvania, USA, 2010–2012
Source: Emerg Infect Dis. 2019 Mar;25(3):473–81. doi: 10.3201/eid2503.180336 (PMC6390762; doi:10.3201/eid2503.180336)
Supplement: Appendix — Whole-genome sequencing and single-nucleotide variant–based phylogenetic analysis of Mycobacterium avium from community and household water, suburban Philadelphia, Pennsylvania, USA, 2010–2012. [file 18-0336-Techapp-s1.pdf]

DOI: https://doi.org/10.3201/eid2503.180336

Mycobacterium avium in Community and Household Water, Suburban Philadelphia, Pennsylvania, USA, 2010–2012

Appendix

Whole-Genome Sequencing and SNV–based Phylogenetic Analysis

The sequenced isolates represented four VNTR types as well as the B1 and B2 subtypes of VNTR 37a (Appendix Figure and Appendix Table 2). For the predominant VNTR 14a group, sequencing included all 10 respiratory specimens plus 19 isolates recovered from 14 households. Selection of isolates for VNTR 36 (n = 5) and VNTR 22 (n = 2) were representative, but less comprehensive. The B1 and B2 subtypes of genotype 37a (n = 4) were included to examine the implication of 3′ *hsp65* gene differences among strains with identical VNTR and ITS profiles.

SNVPhyl analysis identified 26,871 variable sites across the core set of 4.3 million nucleotide positions (Appendix Table 4). With the exception of the biologic replicates, which were identical by SNV analysis, all other isolates had unique SNV profiles. In general, isolates of the same VNTR type were more similar than those of different VNTR types (Appendix Figure). However, SNV analysis did split the twenty–nine VNTR 14a isolates into three distinct clusters (Appendix Figure, panel A). Within each cluster, the distance between any two isolates was <95 SNVs, but the distance between separate clusters was >600 SNVs (Appendix Figure, panel B). Two clusters of VNTR 36, separated by ~225 SNVs, were also observed (Appendix Figure, panel C). Despite their identical VNTR and ITS profiles, the distance between the 37a *hsp65* B1 and B2 subtypes was ~7354 SNVs. SNV analysis revealed household–associated clustering of isolates. Distances for VNTR–matched plumbing isolates from the same household were <60 SNVs (range 2–59).

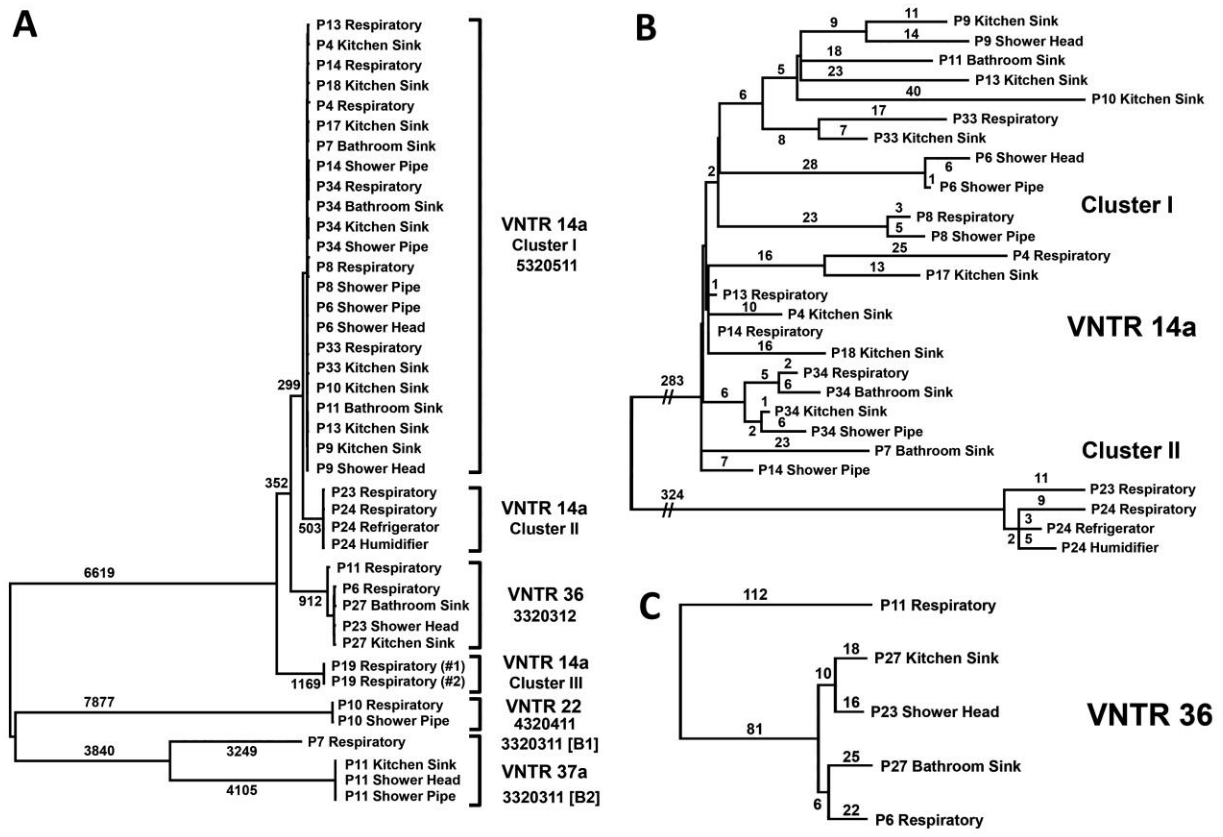

Appendix Figure. Phylogeny of *M. avium* isolates. A) Core SNV analysis provides higher resolution than the combination of VNTR, ITS and *hsp65*-based typing and splits the 29 isolates of the predominant VNTR 14a group into 3 distinct clusters (I– III). B) Analysis of VNTR 14a (subtypes I and II) strains reveals that some respiratory and household isolates differ by <15 core SNVs (e.g., P8, P14, P24, P34). C) Genomic diversity among VNTR 36 strains. Phylogenetic reconstruction was performed using neighbor-joining methods and the 26871 core SNV positions revealed by whole genome analysis of 40 *M. avium* isolates. Trees are not drawn to scale, but branch lengths (number of single nucleotide differences) are shown.
